# Estimating Crop Area at County Level on the North China Plain with an Indirect Sampling of Segments and an Adapted Regression Estimator

**DOI:** 10.3390/s17112638

**Published:** 2017-11-16

**Authors:** Qinghan Dong, Jia Liu, Limin Wang, Zhongxin Chen, Javier Gallego

**Affiliations:** 1 Department of Remote Sensing, Flemish Institute of Technological Research, 2400 Mol, Belgium; 2 Institute of Agricultural Resources and Regional Planning, Chinese Academy of Agricultural Sciences, Beijing 100081, China; liujia06@caas.cn (J.L.); wanglimin01@caas.cn (L.W.); chenzhongxin@caas.cn (Z.C.); 3 Joint Research Centre, The European Commission, 21027 Ispra, Italy; Javier.GALLEGO@ec.europa.eu

**Keywords:** crop area, remote sensing image classification, area frame sampling, stratification, regression estimator

## Abstract

Image classifications, including sub-pixel analysis, are often used to estimate crop acreage directly. However, this type of assessment often leads to a biased estimation, because commission and omission errors generally do not compensate for each other. Regression estimators combine remote sensing information with more accurate ground data on a field sample, and can result in more accurate and cost-effective assessments of crop acreage. In this pilot study, which aims to produce crop statistics in Guoyang County, the area frame sampling approach is adapted to a strip-like cropping pattern on the North China Plain. Remote sensing information is also used to perform a stratification in which non-agricultural areas are excluded from the ground survey. In order to compute crop statistics, 202 ground points in the agriculture stratum were surveyed. Image classification was included as an auxiliary variable in the subsequent analysis to obtain a regression estimator. The results of this pilot study showed that the integration of remote sensing information as an auxiliary variable can improve the accuracy of estimation by reducing the variance of the estimates, as well as the cost effectiveness of an operational application at the county level in the region.

## 1. Introduction

Much research has been conducted in the field of crop mapping and area assessment using satellite image classification. Most often, using high-resolution images such as those generated by the sensors on Landsat satellites, including Landsat 5 Thematic Mapper (TM), Landsat 7 Enhanced Thematic Mapper Plus (ETM+) and Landsat 8 Operational Land Imager (OLI), each pixel is assigned to a specific class of land use or crop [1]. Other methods use an approach of soft or sub-pixel classification, often with coarser spatial resolution images, to increase the geographic coverage and temporal resolution of analysis [2,3]. The classification of spatial objects, which are often called segments, has also become popular, especially for agricultural areas [4].

In the early times of satellite image analysis, crop mapping and crop area estimation were not very well distinguished. Land cover area estimation, and in particular crop area estimation, was considered to be a direct spin-off of classified images that could be obtained by simply counting pixels classified into a particular class [5]. Image analysts became soon conscious that pixel counting generally introduces a bias, and that correcting this bias requires a large amount of field data from a properly sampled set of locations. The most frequent approaches to combine field data with classified images to correct the bias of pixel counting are the calibration and regression estimators [6,7,8,9]. The Group on Earth Observations (GEO) published a more generic discussion of area estimation issues from the remote sensing perspective [10].

The bias of area estimates by pixel counting is related to the inaccuracy of image classification, and can be estimated from the difference between the commission and omission errors for a given class [11]. An important issue that has been studied less is the margin for subjectivity of an area estimation by pixel counting: if the analyst has an idea of the reasonable area of each land cover class (crop, for example), they can tune the image classification parameters, the training sample composition, or the outlier cleaning process to have a pixel counting estimate close to the a priori belief [12]. This type of bias or discrepancy was already reported in other contexts, such as the Activity B “Rapid crop area change estimates at level of European Union” within the European Monitoring Agricultural Resources (MARS) project [13]. It is also well known in the agricultural statistics community that the remote sensing imagery generally gives sound crop area estimates only when it is used together with ground statistical surveys [14].

In this study, the information derived from remote sensing is used both for the design of the sampling frames, and for the final area estimation. First, the satellite images were used to perform a stratification from which non-agricultural areas were excluded from ground survey. In a later stage, the classification of satellite images was included as an auxiliary variable for a regression estimator. On the other hand, the area frame sampling is adapted to the complex landscape of the North China Plain.

**Figure 1:**
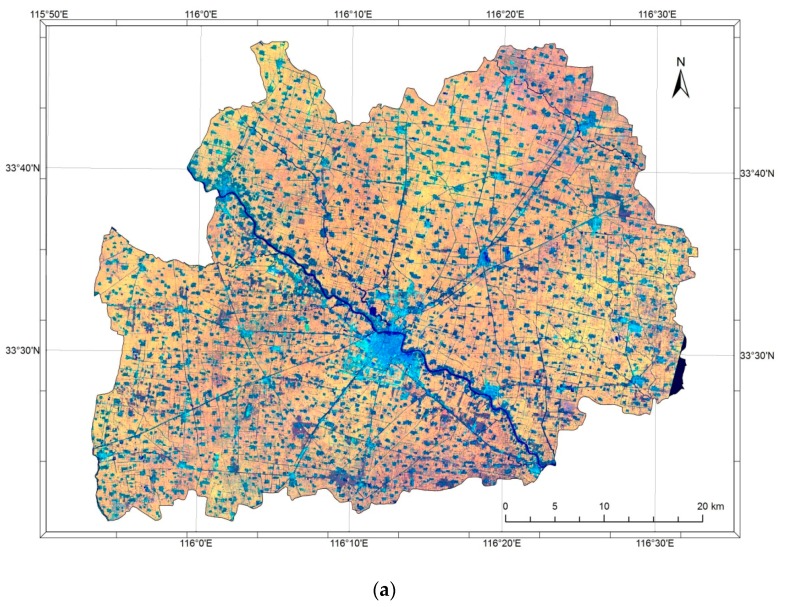

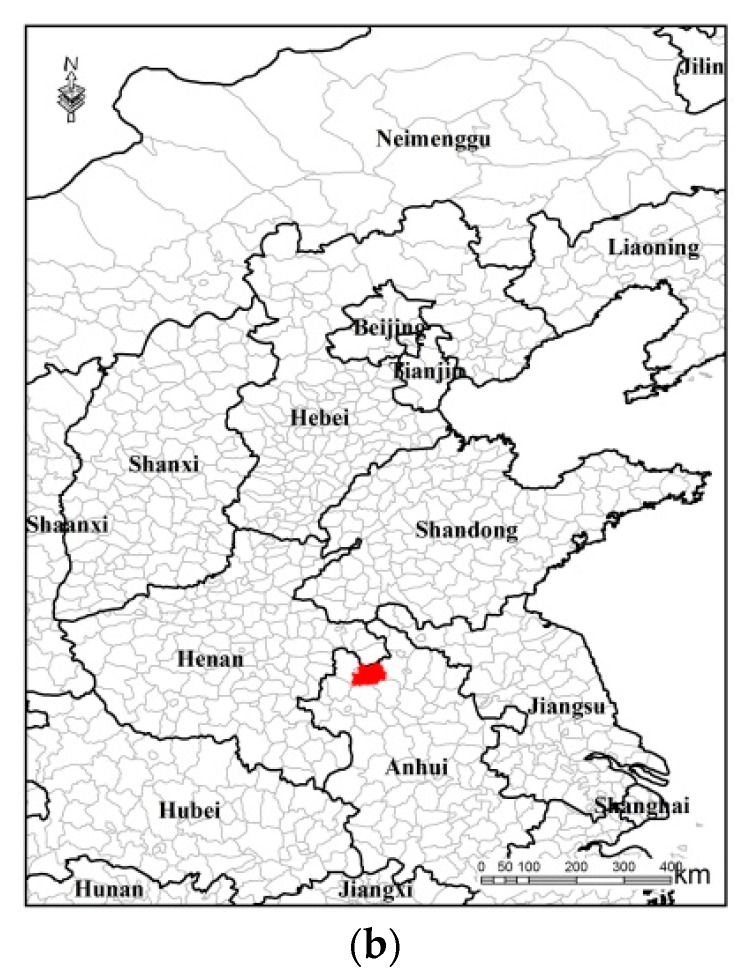
Guoyang County (**a**) is located in the north of Anhui province, and on the south edge of the North China Plain (**b**).

The study was carried out in 2013 in the county of Guoyang, on the southern edge of the plain, in Anhui province (Figure 1). The official statistics data of the year 2012 showed that the county counted 1,545,908 inhabitants within a surface of 210,700 hectares [15]. However, the county border vector generated a figure for the total area of about 214,880 hectares. Similar to elsewhere on the North China Plain, the predominant cropping system consists of two growth seasons for each calendar year, with winter wheat followed by more diversified summer crops, include soybean, maize, cotton, and other vegetables. Although the local government publishes the official crop areas statistics each year, much caution should be paid to these figures, as the planting areas outside of the administrative regulation, such as river beds, are not included in the official statistics. These areas constitute a non-negligible part of the sowing area (up to 20%).

## 2. Materials and Methods

### 2.1. Data

The high resolution images used in this study are two scenes of RapidEye at 5 m resolution, registered on 5 and 13 August 2013, respectively.

The statistical data were acquired from the local statistical office of the Guoyang County government.

### 2.2. Area Frame Sampling—Stage 1: Stratification

Area frame sampling is defined on the geographic space. The units of an area frame can be points, transects (lines of a certain length), or pieces of territory, which are often named segments. When the units of the frame are points, the survey may be called a point survey.

The area frame sampling in this study was carried out in two stages: stratification and segment survey. Prior to the ground survey, a stratification step is performed to separate the agricultural stratum from other strata.

**Figure 2:**
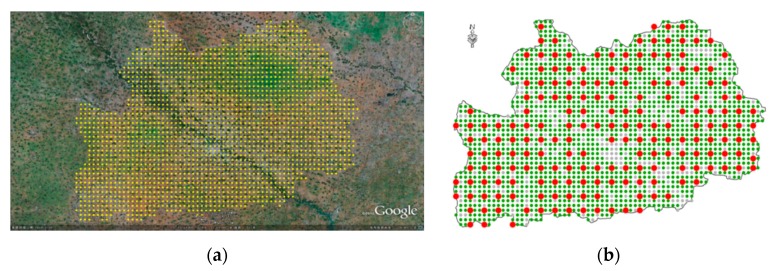
A grid of 0.01° was overlaid on the Google Earth imagery (**a**). There are 2074 grid points within the boarder of the Guoyang County. The sub-sampling of the agricultural stratum was carried out in a systematic way. Two hundred and two grid points plot were thus selected for field survey to assess the crop proportions in this sample/grid point (**b**).

The stratification starts with a regular grid of 0.01° × 0.01° overlaid to Google Earth in the study region most often composed by 2012 and 2013 imagery (Figure 2a).

The grid points located within the border of Guoyang County were stratified by photo-interpretation into two strata: non-agricultural and agricultural.

The agricultural stratum is subsampled for survey purpose. The number of points to be surveyed is determined by the available resources. In this study, 202 points that were stratified to the agricultural stratum were selected for survey (Figure 2b).

A printout of Google Earth with an approximate scale 1:10,000 was used for guiding the ground survey.

### 2.3. Area Frame Sampling—Stage 2: Ground Survey

**Figure 3:**
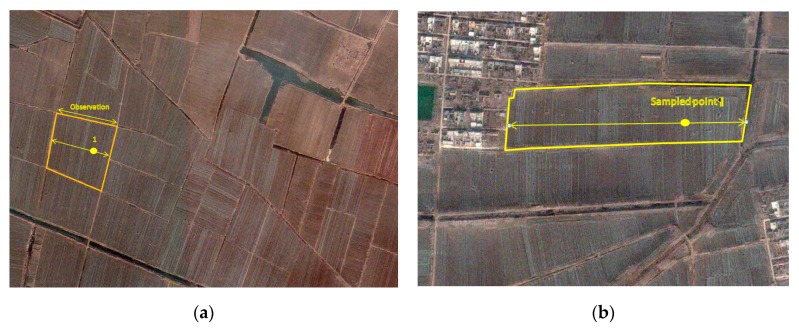
The crop proportion of a particular grid/sample point was assessed by expanding the point to a segment according to the physical boundaries of the field plot harboring the grid/sample point. The proportion of each crop was assessed by global positioning system (GPS) measuring on one border of the plot, perpendicular to the field strips (**a**). The expanded segment can be located on the edge of the agricultural stratum (**b**).

The region dispays a very characteristic cropping landscape, where fields of a few hectares are divided into thin stripes and cultivated by different households. Due to this stripe-shaped pattern, the sampling procedure is adapted, starting with 202 points. The points are then expanded to generate “segments” (Figure 3). A segment is thus conceived as a set of parallel stripes that does not straddle any permanent linear element (dirt road, hedge, etc.). The proportion of the targeted crop in segment is assessed by the proportion along the transect. The geographical location was performed using a Trimble GeoXT in stand-alone mode.

From a statistical point of view, as only the agricultural stratum is sampled in our approach, we identify below this stratum with the population, and we omit for the moment the stratum sub-index. A segment *i* with area *A_i_* is selected with probability $\pi_{i}=\frac{n\;A_{i}}{D}$, where *D* is the total area and *n* = 202 is the sample size. The usual Horvitz–Thompson estimator for the total area of crop *c* with unequal sampling probability is:

(1) $${\hat{Y}}_{c}=\sum_{i}\frac{y_{i}}{\pi_{i}}=D\sum_{i}\frac{y_{i}}{n\;A_{i}}=D\sum_{i}\frac{p_{i}}{n}$$

where *y_i_* is the area of crop *c* in segment *i,* and *p_i_* is the proportion. Thus, the unbiased unequal sampling probability estimator using the area of each crop in each segment coincides with the estimator based on the proportions and assuming equal probability. Therefore, estimators based on proportions are easier to manage, including regression estimators.

This ground survey will generate crop area estimates for each of the surveyed segments (y in the Section 2.5) and its mean ($\bar{y}$). The estimation is obtained by measuring the width of strips cultivated with one crop type.

### 2.4. Image Classification

The RapidEye scenes were interpreted using the maximum likelihood classifier [16]. The ground truth data derived from the survey segments were split into two sets according to a ratio of 1:7 in a systematic sampling way:

- according to the field numbering in a set of eight plots, the first seven plots are used to train the classifier;
- the eighth plot of the set was assigned to the validation dataset to assess the accuracy of the classifications.

The image classification will generate the values for the variables used in the subsequent regression estimation analysis (Section 2.5): the variable p represents the crop area proportion in each of the 202 segments, its mean is ($\bar{p}$), and ${\bar{p}}_{\mathrm{pop}}$ is the percentage of the pixels classified as the target crop in the entire agriculture stratum.

### 2.5. Regression Estimator

The regression estimator improves the accuracy of area estimates by adjusting the estimate of mean and reducing the variance [17,18].

In this study:

(2) $${\bar{y}}_{\mathrm{reg}}=\bar{y}+b\left({\bar{p}}_{\mathrm{pop}}-\bar{p}\right)$$

where ${\bar{y}}_{\mathrm{reg}}$ is the regression estimate for a target crop area; $\bar{y}$ is the area mean derived from the ground survey for the target crop; ${\bar{p}}_{\mathrm{pop}}$ is the proportion of pixels classified as the target crop in the agriculture stratum of the county; and $\bar{p}$ is the average proportion of pixels classified as the target crop in the surveyed segments. Also, b is the slope of the regression between y (the target crop proportion in each segment obtained from the ground survey) and p (the target crop proportion in each segment generated by image classification).

For large random samples (202 in this study), the variance of the regression estimator $\mathrm{var}\left({\bar{y}}_{\mathrm{reg}}\right)$ can be approximated by:

(3) $$\mathrm{var}\left({\bar{y}}_{\mathrm{reg}}\right)=\mathrm{var}\left(\bar{y}\right)\left(1-R_{\mathrm{py}}^{2}\right)=\frac{1}{n}\mathrm{var}\left(y\right)\left(1-R_{\mathrm{py}}^{2}\right)$$

where $R_{\mathrm{py}}^{2}$ is the coefficient of determination for the regression.

The relative efficiency of the regression estimator ($\eta_{\mathrm{reg}}$) can be approximated as the ratio of the estimator’s variances, and defined as:

(4) $$\eta_{\mathrm{reg}}=\frac{\mathrm{var}\left(\bar{y}\right)}{\mathrm{var}\left({\bar{y}}_{\mathrm{reg}}\right)}$$

Relative efficiency can be interpreted as the relative sample size of the original ground survey required to achieve the certainty of the estimates derived from the regression estimation.

## 3. Results

### 3.1. Stratification and Segment Survey

As described above, two strata were defined: (1) agriculture (arable land) and (2) non-agriculture (urban, artificial, water). In this study case, all of the grid points could be exactly photo-interpreted with a priori knowledge. For regions in which ambiguity of interpretation is a relevant problem, a third stratum, “doubts”, can be considered.

Each of the 2074 grid points in total was assigned to one of two strata. Out of all of the points, 1502 points, or 72.42% (155,616 hectares) were identified as belonging to the stratum “arable land”, and 572 points or 27.58% (59,264 hectares) were interpreted as belonging to the non-agricultural stratum. In the next stage of the sampling, 202 points sampled in the agricultural stratum were expanded to segments, as described in Section 2.3 and surveyed in August 2013. The results of the survey for these expended segments are summarized in Table 1, where the average size of 202 surveyed segments is also indicated.

### 3.2. Image Classification

**Figure 4:**
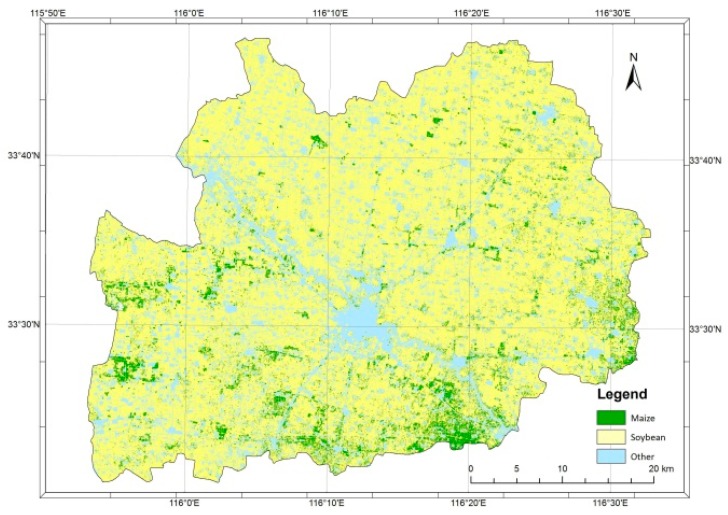
Classification using two scenes of RapidEye imagery.

Figure 4 and Table 2 show the results of image classification using two registrations of the RapidEye imagery. They illustrated that the crop land covers 84.5% of the classified image, with soybean and maize sharing 85.29% and 14.64% of the total crop land area, respectively. It is worth noting that an estimate of the agriculture area generated by photo-interpretation is somehow lower (72%). The results also reveal the area percentages of maize and soybean classes in the 202 survey segments (Table 2).

Figure 4 also reveals that maize appears to be under-cultivated in this county in comparison with the neighboring counties, and is more commonly found on the southeastern part of the county, especially near the border with Mengcheng County [19].

The class “other crops”, which includes various crops such as cotton and peanuts, is a very small proportion of what is cultivated in this county. However, the image classification provides a still smaller area for this class, as it is marginally represented.

The validation results of the classifications were carried out using a confusion matrix (data not shown). The overall accuracy was found to be high—around 95%—which was contributed mostly by the highly dominant soybean crop in the county.

### 3.3. Regression Estimator

**Figure 5:**
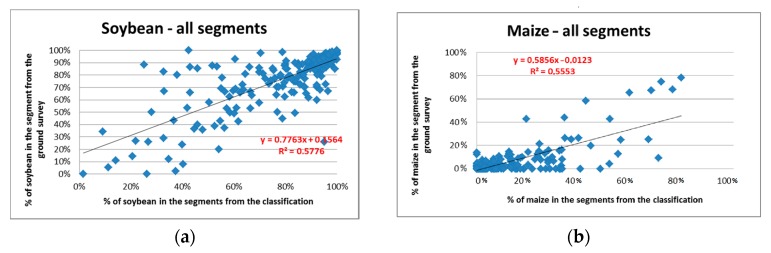
Regressions of crop proportions for the soybean (**a**) and maize (**b**) derived from 202 surveyed segments against those derived from the classification for the same segments.

The regressions between the percentages of crops in the 202 sampled segments derived from the field survey and those computed from image classification for the same segments are illustrated in Figure 5.

The $R_{\mathrm{py}}^{2}$ of the regressions between the crop proportions (from the 202 segments) derived from the ground survey and from the classification are 0.58 and 0.56 for the classes “soybean” and “maize”, respectively. The standard deviations (SD) of the estimates obtained with the regression estimator are subsequently reduced from 1.72 to 1.14 for maize and 0.34 to 0.22 for soybean, when the image classification is used as an auxiliary variable (Table 3).

For example, the regression estimated soybean percentage in Guoyang County in 2013 can be deduced:

(5) $${\bar{y}}_{reg,\;soybean}=91.58+0.78\times (85.29-88.36)=89.02$$

The relative efficiency of the regression estimation approach for soybean and maize becomes 2.4 and 2.5, respectively, when Equation (4) is applied.

## 4. Discussion

In summary, this study tries to demonstrate the efficiency of the regression estimator approach in estimating the crop areas at a county level. The study region was located in Guoyang County on the North China Plain. The contribution of remote sensing was made on two levels. (1) It helped in area frame stratification, therefore in optimizing the sampling design prior to the ground survey. (2) Remote sensing was incorporated as an auxiliary variable in the analysis of regression estimation by means of image classification, and therefore improved the precision of estimates by reducing the variance of estimation.

More specifically, the area proportions for soybean and maize in the surveyed segments were regressed against those derived from the image classification. The regression estimator allowed adjusting the crop area estimates while reducing the variance of these estimates by a factor between 2.4 and 2.5 in this study. An efficiency value of 2.5 means that the accuracy obtained from sampling 202 segments corrected with remote sensing will be approximately equivalent to the accuracy obtained from sampling 505 segments by ground survey only. From a cost efficiency point of view, the approach will greatly reduce the ground survey costs, whereas relatively low costs for image acquisition and analysis need to be added.

As the use of high resolution imagery (such as Sentinel 2) becomes more generalized and data costs drop sharply, the application by combining ground survey data and satellite information will further show both its scientific soundness and cost-effectiveness in agricultural statistic surveys, especially in remote areas or parceled farming landscapes, such as those on the North China Plain.

## Figures and Tables

**Table 1:** Statistics on the 202 segment survey.

	Maize	Soybean	Other Crops	Non-Agriculture
Surface proportion (%)	6.21%	91.58%	2.01%	0.20%
Standard deviation (%)	1.85%	0.34%	7.70%	8.25%
Average segment size (ha)	4.69			

**Table 2:** Percentage of each class derived from the image classification.

	% Area of the County	% of Crop Land Area	% of Classified Pixels in Surveyed Segments
Soybean	72.07	85.29	88.36
Maize	12.37	14,64	10.32
Other crops	0.06	0.07	n/a
Other land use	15.50	n/a	n/a

**Table 3:** Crop area proportions derived from a ground survey, image classification, and the regression estimator. SD = standard deviation.

	Maize	Soybean
Mean of the area percentage from surveyed segments (and its SD)	6.21% (1.72%)	91.58% (0.34%)
Regression slope (b) and coefficient of determination ($R_{\mathrm{py}}^{2}$)	0.59 (0.56)	0.78 (0.58)
Area percentage from classification in the agriculture stratum	14.55%	85.29%
Mean of the area percentage from classified segments	10.32%	88.36%
Regression estimates in the arable stratum (and its SD)	8.76% (1.14%)	89.20% (0.22%)
Relative efficiency of remote sensing	2.5	2.4
Number of ha in the county (assuming 155,616 ha arable area)	16,558 ha	138,809 ha
